# Work-related musculoskeletal risk factors in medical laboratory students: A cross-sectional insights from a RULA-based assessment with lifestyle correlates

**DOI:** 10.1038/s41598-026-41498-z

**Published:** 2026-02-27

**Authors:** Mshari Alghadier, Abdulaziz Alsubaie, Abdulmajeed Alrabie, Khalid Alquraini

**Affiliations:** Department of Health and Rehabilitation Sciences, Prince Sattam bin Abdulaziz University, Alkharj, 11942 Saudi Arabia

**Keywords:** Ergonomics, Laboratory, Musculoskeletal Diseases, Posture, Quality of Life, Diseases, Health care, Health occupations, Medical research, Risk factors

## Abstract

Work-related musculoskeletal risks in student laboratory settings are under-characterized. We conducted a cross-sectional study of 31 healthy male medical laboratory students to quantify upper-limb ergonomic risk using the Rapid Upper Limb Assessment (RULA) and examine associations with physical activity (International Physical Activity Questionnaire—Short Form), sleep quality (Pittsburgh Sleep Quality Index), quality of life (WHOQOL-BREF), and anthropometry. Standardized 5-min task videos were independently scored by three trained assessors. RULA scores clustered at mid action levels, indicating investigation and possible modification for most and prompt action for a notable minority. Across physical-activity strata, between-group differences for upper-arm posture (χ^2^(2) = 6.07, *p* = 0.04), the upper-limb composite Score A (χ^2^(2) = 7.69, *p* = 0.02), and the adjusted upper-limb composite Score C (χ^2^(2) = 6.35, *p* = 0.04), with the high-activity group generally exhibiting more favorable scores. In multivariable ordinal models, higher BMI predicted worse wrist-posture categories (OR = 1.33, 95% CI [1.02–1.73], *p* = 0.03), whereas PSQI, overall QoL, and IPAQ contrasts were not independent predictors of Score A or upper-arm posture. These findings identify upper-limb postural load as the predominant ergonomic concern and suggest anthropometric fit and movement behaviors as actionable targets. Given the small, single-site, male-only sample, results are preliminary; curriculum-embedded ergonomic adjustments (bench/eyepiece height, forearm support, micro-breaks) warrant evaluation in larger, mixed-gender, multi-site studies.

## Introduction

Work-related musculoskeletal disorders (WRMSDs) are among the most prevalent occupational health conditions globally, driving substantial productivity loss, absenteeism, and long-term disability^1^. Collectively, musculoskeletal disorders affect over 1.7 billion people worldwide, making them a leading cause of disability^2,3^. These disorders often result from repetitive tasks, challenging postures, prolonged static positions, and inadequate ergonomic conditions. While WRMSDs are well-documented in clinical and industrial settings, limited attention has been given to their occurrence among students engaged in laboratory-based academic programs^4,5^.

Prior studies indicate that overall WRMSD prevalence among medical laboratory professionals ranges from 40% to 60% worldwide, with the neck most frequently affected (18–78%)^6^. Medical science laboratory students are frequently exposed to ergonomically demanding tasks such as microscopy, pipetting, and sample handling—activities that mirror the physical demands of professional laboratory work^7,8^. These tasks often involve sustained upper limb activity and non-neutral postures, which can predispose students to early onset of musculoskeletal symptoms. As students’ progress through their training, the cumulative exposure to poor ergonomic practices may increase their susceptibility to WRMSDs^9^.

Recent findings suggest that many students adopt awkward neck and upper back postures when using microscopes or lab benches, placing them at risk for WRMSDs early in their careers^10–12^. Musculoskeletal health in laboratory settings is multifactorial, arising from interactions among workstation geometry and task demands, exposure dose (duration, repetition, force, contact stress), organizational conditions (workload, scheduling, breaks, training), and individual characteristics (anthropometry, prior symptoms, fitness, sleep).

Beyond the physical workspace, lifestyle factors shape tolerance to static loading and motor control during precision tasks. Physical activity (PA) is any skeletal-muscle–produced movement that increases energy expenditure across leisure, transport, domestic, and occupational domains, at light, moderate, or vigorous intensities^13,14^. It is generally associated with greater musculoskeletal resilience, including strength, flexibility, joint mobility, and postural stability. By contrast, sedentary behavior is linked to poorer postural endurance and a higher vulnerability to overuse injury^15–18^. Importantly, excessive or poorly structured PA may increase risk—particularly when external loads are high, movements are repetitive, recovery is insufficient, or technique is suboptimal. Observational studies show higher rates of back and upper-limb complaints in activity patterns characterized by heavy/repetitive loading and prolonged static tasks, underscoring dose–response and context dependency^19,20^.

Sleep quality plays a pivotal role in musculoskeletal health and the body’s ability to recover from physical strain^21,22^. Among university and medical students, the prevalence of poor sleep quality typically ranges from 19 to 58%, with several cohorts reporting ≥ 50%^23,24^. Poor sleep is associated with fatigue, reduced pain tolerance, and impaired neuromuscular coordination, potentially heightening WRMSD risk during repetitive or sustained tasks typical of laboratory work^21^. Ergonomic exposures can also increase pain and discomfort that disrupt sleep, creating a bidirectional cycle between sleep disturbance and ergonomic risk^25–29^. Addressing both sleep quality and ergonomic risk factors is essential for developing comprehensive strategies to prevent WRMSDs and promote overall well-being among individuals in physically demanding environments^30^.

Quality of life (QoL) is a multidimensional concept encompassing an individual’s physical health, psychological state, social relationships, and interaction with their environment^31^. Among university students, particularly in demanding programs such as medical laboratory sciences, QoL is shaped by lifestyle, academic pressure, and laboratory demands. Lower QoL correlates with higher stress, fatigue, and musculoskeletal complaints and may reduce engagement in protective behaviors (e.g., exercise, ergonomic practices), whereas higher QoL aligns with healthier choices, better coping, and adherence to preventive self-care that can mitigate ergonomic stressors^32–35^.

The Rapid Upper Limb Assessment (RULA) is a widely used ergonomic screening tool designed to evaluate postural risk factors for the neck, trunk, and upper limbs^36^. It is especially useful in environments where individuals engage in sedentary yet repetitive activities. While numerous studies have applied RULA in clinical and industrial contexts, its application in academic laboratory settings—especially among students—remains limited^37,38^. While posture-focused tools such as RULA capture segmental risk during task performance, WRMSDs in laboratory settings are multifactorial. Explicit assessment of the macro-ergonomic domains—alongside posture—would enable a more complete understanding of risk pathways and more effective mitigation strategies in student laboratories. Because RULA scores primarily reflect observed posture, they are expected to be strongly shaped by task demands and workstation geometry. Accordingly, the present study does not aim to infer causal effects of individual-level exposures, but rather to evaluate whether variability in RULA-defined postural risk under a standardized task protocol is associated with selected individual characteristics. Determining causality and underlying mechanisms (including potential fatigue-related effects) will require experimental and/or longitudinal studies incorporating objective exposure metrics and controlled manipulation of task and workstation parameters.

To address the gap in student laboratory ergonomics, we examined whether individual-level characteristics are associated with variability in task-level postural risk measured under a standardized laboratory protocol. While workstation geometry and task demands are expected to be the primary determinants of RULA scores, individuals may differ in their habitual postural strategies and movement patterns when performing the same standardized activities. We therefore quantified RULA-defined postural risk during core laboratory tasks and evaluated its cross-sectional associations with habitual PA, sleep quality, body composition, and health-related QoL. We prespecified that (i) higher PA, better sleep quality, and higher QoL would be associated with lower RULA scores, and (ii) higher BMI/weight would be associated with higher RULA scores, noting that this anthropometric relationship is consistent with established ergonomics evidence. We further acknowledge that determining causality and isolating mechanisms will require experimental or longitudinal designs integrating workstation audits and exposure metrics (e.g., duration, repetition, force) as well as training and organizational determinants.

## Methods

### Study design and participants

This was an observational, cross-sectional study conducted between October and December 2023 to evaluate WRMD risk among male laboratory students at Prince Sattam bin Abdulaziz University, Saudi Arabia. Data were collected using a standardized laboratory task protocol to minimize variability in task content and timing across participants. No experimental manipulation was performed; therefore, analyses were limited to assessing associations rather than causal inference. Participants were recruited via university email announcements and posters displayed in common areas on the male campus. A convenience sampling method was utilized to recruit 31 male students from the Laboratory Science Department. A male-only cohort was used due to gender-segregated facilities and the need for video recording during task performance, which limited access to female labs in the study period. This sampling strategy constrains external validity—generalization to female or mixed-gender cohorts is limited—but restricting participation to one gender reduces heterogeneity and gender-related confounding, thereby enhancing internal validity. Accordingly, we prioritize effect sizes and 95% confidence intervals (CI) and interpret the findings as preliminary. A formal a priori power calculation was not feasible given timetable constraints and the exploratory objective. We targeted a feasible cohort of ~ 30–35 participants to establish preliminary estimates and precision for key effects. To mitigate small-sample bias, we: (i) report effect sizes (ε^2^ for group tests; ORs with 95% CIs for ordinal models), (ii) apply Huber–White robust standard errors, and (iii) run non-parametric/quantile sensitivity analyses where assumptions are not met. This study adheres to the STROBE (Strengthening the Reporting of Observational Studies in Epidemiology) recommendations for cross-sectional studies^39^.

### Inclusion and exclusion criteria

Inclusion criteria included being male laboratory students enrolled in the Laboratory Science Department, aged between 19 and 25 years, and providing voluntary informed consent. Exclusion criteria comprised students with known musculoskeletal disorders or injuries diagnosed by a medical professional, students who had undergone surgery affecting musculoskeletal health within the previous six months, and students unwilling or unable to provide informed consent.

### Data collection instruments

#### Rapid upper limb assessment (RULA)

The RULA is an observational method widely utilized to evaluate biomechanical and postural risks associated with upper limb disorders. RULA scoring categorizes risk levels and highlights priorities for ergonomic interventions^36^. It is widely recognized as a reliable observational tool and has shown good intra- and inter-rater reliability, making it effective in identifying ergonomic risks and guiding intervention strategies^40^. Published studies generally report good–excellent rater agreement for RULA when assessors are trained and scoring protocols are standardized, intra- and inter-rater intraclass correlation coefficient (ICC) values 0.90–0.94 and 0.89–0.93, respectively^41^.

The RULA assessment involves assigning a numerical score to the posture of the upper arms, lower arms, and wrists (Score A), as well as to the posture of the neck, trunk, and legs (Score B). Additional points are then added for factors that place extra strain on the musculoskeletal system, such as repetitive movements, sustained static postures, and force exertion. These adjustments result in Score C (Score A + muscle use and force scores for Group A) and Score D (Score B + muscle use and force scores for Group B). An algorithm then combines these values to produce a Grand RULA (1–7) maps to Action Levels 1–4 (1–2 acceptable; 3–4 further investigation; 5–6 investigation/changes soon; 7 immediate change).

#### International physical activity questionnaire–short form (IPAQ-SF)

International Physical Activity Questionnaire–Short Form (IPAQ-SF) is a validated self-report questionnaire designed to quantify PA levels during the last 7 days, distinguishing between low, moderate, and high PA, thus providing essential lifestyle context for musculoskeletal health research^42^. Categories follow standard IPAQ-SF criteria: low = does not meet moderate/high thresholds (typically < 600 MET-min/week); moderate = ≥ 600 MET-min/week (e.g., ≥ 3 days vigorous ≥ 20 min/day, or ≥ 5 days moderate/walking ≥ 30 min/day, or any combination totaling ≥ 600 MET-min/week); high = ≥ 1500 MET-min/week from vigorous activity on ≥ 3 days or ≥ 3000 MET-min/week from any combination on ≥ 7 days. It demonstrates excellent test-retest reliability and criterion validity, making it suitable for assessing the association between PA and musculoskeletal conditions^43^. The validated Arabic version of IPAQ-SF was used in this study^44^.

#### World health organization quality of life-BREF (WHOQOL-BREF)

World Health Organization Quality of Life-BREF (WHOQOL-BREF) is a standardized 26-item questionnaire widely validated across diverse cultural settings. It demonstrates robust psychometric properties, including strong internal consistency, reliability, and validity thus providing a comprehensive assessment of QoL relevant to musculoskeletal health outcomes^45^. In Arab populations, the Arabic WHOQOL-BREF shows high internal consistency (α ≥ 0.70) and adequate test–retest reliability with item-level and domain-level validity indices meeting recommended thresholds in a large general-population study from Kuwait^46^. Additional Arabic validations (e.g., caregivers in Jordan) report Cronbach’s α ≥ 0.70 with item–domain correlations ≥ 0.40 and confirm the canonical four-domain solution^47^. In this study, we used the validated Arabic WHOQOL-BREF^46,47^.

It comprises four domains: physical health (7 items), psychological health (6 items), social relationships (3 items), and environmental health (8 items). In addition, it includes two general items addressing overall QoL and general health. Each item is rated on a five-point ordinal scale ranging from 1 to 5, with higher scores indicating a better perceived QoL. These raw scores are then transformed linearly to a 0–100 scale^48,49^. The physical health domain assesses aspects such as mobility, daily activities, functional capacity, energy, pain, and sleep. The psychological health domain encompasses measures of self-image, negative and positive feelings, self-esteem, mental well-being, learning ability, concentration and memory, religious beliefs, and overall mental status. The social relationships domain evaluates personal relationships, perceived social support, and satisfaction with sexual life. Finally, the environmental health domain addresses factors including financial resources, personal safety, access to health and social care services, quality of the physical living environment, opportunities for acquiring new skills and knowledge, recreational possibilities, and broader environmental conditions such as noise, air quality, and transportation.

#### Pittsburgh sleep quality index (PSQI)

Pittsburgh Sleep Quality Index (PSQI) is a reliable and validated self-administered tool that measures subjective sleep quality and disturbances over a one-month period, producing scores from 0 to 21, with higher scores indicative of poorer sleep quality^50^. The standard diagnostic cut-off is a global score > 5, which indicates poor sleep; scores 0–5 is typically considered good sleep quality. It consistently exhibits good internal consistency, high test-retest reliability, and strong construct validity, making it an ideal instrument to explore potential associations between sleep quality and musculoskeletal disorders^51^. Among patients with primary insomnia, the PSQI global score demonstrated excellent test–retest reliability (*r* = 0.87). Convergent validity with sleep logs was strong, whereas correlations with polysomnography were lower. In the same sample, the conventional > 5 cut-point classified poor sleepers with 98.7% sensitivity and 84.4–84.8% specificity^52^. In Arabic-speaking contexts, validation studies report acceptable reliability and validity: an Arabic PSQI showed Cronbach’s α = 0.77 with good discriminative and construct validity, and no floor/ceiling effects in an adult sample; bilingual Arabic–English versions demonstrate moderate–strong test–retest reliability (ICC 0.63–0.87) with α = 0.65; additional dialectal adaptations (e.g., Tunisian Arabic) report α = 0.60 alongside supportive factor-analytic evidence, collectively supporting use in Arabic-speaking students and clinicians with acknowledgment of dialectal variation^53,54^.

### Procedure and task selection

The study was conducted in the Laboratory Science Department laboratories, College of Applied Medical Sciences, Prince Sattam bin Abdulaziz University. During a standardized briefing, investigators answered all questions to ensure comprehension of procedures and objectives. At the data-collection visit, demographic information (age, height, weight, BMI) was obtained. Participants then performed curriculum-embedded laboratory tasks with high upper-limb and axial demands, pipetting, microscopy, and seated sample handling. These tasks were selected because they (i) occur in > 80% of practical sessions, (ii) require sustained or awkward upper-limb postures and fine-motor control, and (iii) are executed on workstations with consistent configuration across students.

All assessments were performed in standard teaching laboratories equipped with fixed bench height and microscope stations. Chairs were adjustable in height, and participants were instructed to adopt neutral seated posture (feet supported, hips/knees at 90°, forearms supported when feasible). We documented which elements were fixed (bench, microscope eyepiece height relative to bench) and which were adjustable (chair height/footrest). Participants were allowed to adjust chairs to achieve neutral posture; other elements remained fixed to minimize between-participant workstation variance.

For each task, a standardized protocol defined the start/stop events, required body position, hand placement, and tool use (e.g., microscopy with bilateral forearm support; single-channel pipetting using a neutral wrist grip; sample sorting at bench height). Each task was performed for 5 min to capture stable, representative postures under standardized conditions while minimizing posture drift and observer reactivity; this duration was selected for postural characterization rather than to provoke acute fatigue. Consequently, any associations with PA or sleep quality should be interpreted as reflecting baseline participant characteristics rather than short-term fatigue development during the observation period. To promote consistent execution and reduce learning effects, participants received standardized instructions and completed a brief practice period prior to scoring. RULA scoring was conducted within predefined task segments using the same workstation configuration and identical task instructions for all participants; as a posture-based tool, RULA reflects typical posture under these constraints rather than cumulative exposure. Each participant was recorded using a Nikon D5600 digital video camera (Nikon Corporation, Tokyo, Japan) mounted on a tripod at standardized heights and angles to ensure clear, unobstructed views for postural assessment. Video recordings were subsequently reviewed to characterize posture and repetitive movements, with time-stamped annotations created to support scoring using Kinovea software (www.kinovea.org).

To align with worst-case posture assessment, trained assessors extracted four frames per task: three representative frames at 60 s, 180 s, and 300 s, plus one worst-case posture frame identified by visual review. Three assessors scored all frames independently using the RULA procedure (segment positions, muscle-use and force/load adjustments). For each participant, segment and composite RULA outcomes were defined as the maximum (worst-case) score across sampled frames, and disagreements were resolved by consensus. Inter-assessor agreement was evaluated using a two-way random-effects, absolute-agreement ICC for all segment and composite outcomes (Upper arm, Lower arm, Wrist, Neck, Trunk, Legs, Scores A–D, Grand RULA), with a priori thresholds of < 0.50 (poor), 0.50–0.75 (moderate), 0.75–0.90 (good), and > 0.90 (excellent). Inter-assessor reliability was good to excellent across most outcomes (e.g., Grand RULA ICC = 0.86; Score A ICC = 0.82; Score B ICC = 0.79).

**Fig. 1:**
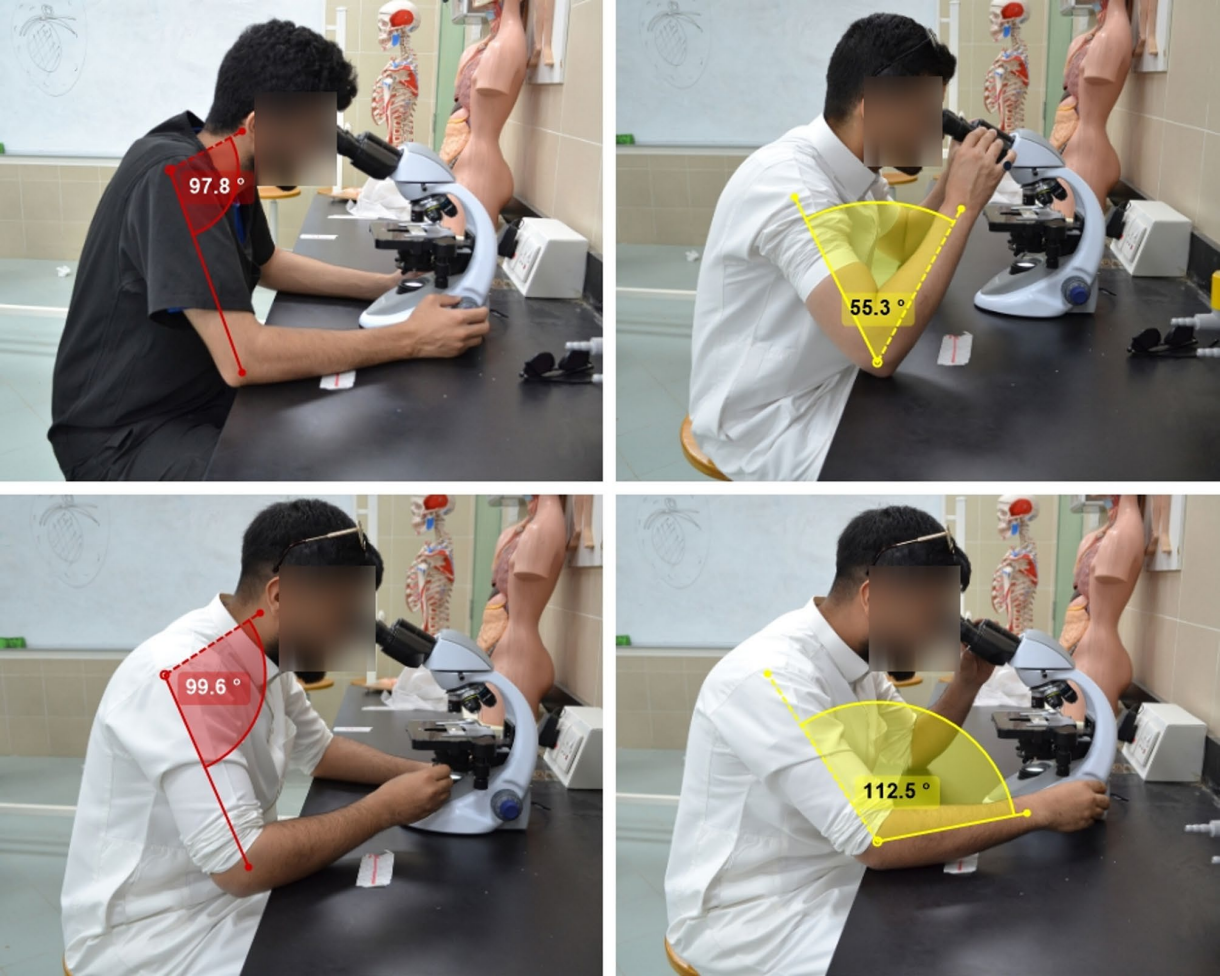
Experimental setup and posture scoring. Videos were acquired using a tripod-mounted camera at fixed height and angle to visualize upper-limb and axial segments. Faces were blurred to preserve anonymity, and all participants provided written informed consent for recording and publication. Bench height, chair settings, and microscope eyepieces are indicated to contextualize posture constraints. Annotations were created with Kinovea (version 2023.1.2; © 2006–2024 Joan Charmant and contributors; www.kinovea.org).

Postures were scored using the RULA tool, and segment scores were computed for the upper arm, lower arm, and wrist, and for the neck, trunk, and legs; composite scores included Score A (upper-limb segments), Score B (neck–trunk–legs), Score C = Score A + muscle-use and force/load adjustments, and Score D = Score B + adjustments. These values were combined to yield a Grand RULA score (1–7) mapped to Action Levels 1–4, which indicate the urgency of corrective action. Immediately after task recording, participants completed the IPAQ-SF, WHOQOL-BREF, and PSQI electronically via a secure platform; anonymity was preserved by replacing identifiers with unique study codes. Figure 1 depicts the experimental setup and task execution.

### Data analysis

All analyses were conducted in R (v2023.6.0–1). Continuous variables are summarized as median (IQR) and mean ± SD; and categorical variables as n (%). WHOQOL-BREF domain scores were transformed to a 0–100 scale following the standard formula from the guideline (raw domain score − 4) × 100/16; higher values indicate better QoL. For descriptives only, domain categories were displayed as tertiles (low, moderate, and high) and inferential analyses used continuous scores. PSQI was scored 0–21 (higher = poorer sleep); we report the global score and, where descriptive categories are shown, poor sleep = PSQI > 5. IPAQ-SF was processed to total MET-minutes/week and categorized per standard thresholds: low (< 600 MET-min/week), moderate (≥ 600), and high (≥ 1500 from vigorous on ≥ 3 days or ≥ 3000 from any combination on ≥ 7 days).

Distributional assumptions were evaluated with Shapiro–Wilk, histograms, and Q–Q plots. Because several outcomes deviated from normality and *n* = 31 was modest, primary group comparisons of RULA outcomes across IPAQ-SF categories used Kruskal–Wallis with Dunn–Holm post-hoc tests; we report ε^2^ effect sizes with 95% CIs. For multivariable analyses, we modeled RULA outcomes using cumulative-link (proportional-odds) ordinal regression, reporting odds ratios (ORs) with 95% CIs. For visualization, regression coefficients were plotted on the log-odds scale (log[OR]) with 95% CIs. To mitigate sensitivity to variance misspecification, we used Huber–White robust standard errors for inference. The proportional-odds assumption was evaluated with score tests; where results were borderline, we performed sensitivity analyses using partial proportional-odds specifications. To limit over-parameterization relative to sample size and reduce collinearity, models were prespecified and parsimonious: BMI (without concurrent inclusion of height or weight in the same model), PSQI global score, and PA contrasts (reference = high PA), with overall QoL included as a single well-being indicator. We quantified multicollinearity using variance inflation factors (VIFs); all VIFs were < 2. Two-sided α = 0.05 defined statistical significance. Given the feasibility-constrained sample, we emphasize precision and effect sizes over dichotomous significance testing. Post hoc, the Kruskal–Wallis tests provided approximately moderate power to detect medium effects. Ordinal regression results are therefore presented as exploratory with conservative interpretation, highlighting ORs with 95% CIs and avoiding strong claims where uncertainty remains substantial. All reported relationships represent associations between participant characteristics and RULA-defined postural risk under standardized conditions.

### Ethical considerations

Ethical approval was obtained from Departmental Ethical Committee, Department of Health and Rehabilitation Sciences, Prince Sattam bin Abdulaziz University, Saudi Arabia (No. RHPT/023/008; date: 01/09/2023) and informed consent was secured from each participant prior to their involvement in the study. This study adhered strictly to the Declaration of Helsinki. Participation was voluntary, and confidentiality and anonymity were maintained throughout the study. Participants were informed of their right to withdraw at any point without any repercussions.

## Results

A total of 31 healthy male participants from Prince Sattam bin Abdulaziz University met the inclusion criteria and were included in the study. The participants’ age ranged from 19 to 25 years old, height ranged from 160 to 188 cm, weight ranged from 48 to 179 kg, while their BMI ranged from 15.6 to 37.5 kg/m^2^. Most of the participants were in the healthy-weight range, with notable proportions in overweight and obese categories and a smaller underweight subgroup. Table 1 summarizes the demographic characteristics of the sample.

**Table 1:** Demographic characteristics of the sample.

Variable	Category	Mean ± SD/*n* (%)
Age, y		21.5 ± 1.5
Height, cm		173.4 ± 6.5
Weight, kg		77.8 ± 26.2
BMI, kg/m^2^		24.9 ± 5.8
Preferred hand	Right	29 (93.5)
	Left	2 (6.5)
BMI category	Underweight	4 (12.9)
	Healthy weight	14 (45.2)
	Overweight	6 (19.4)
	Obese	7 (22.6)
IPAQ-SF	Low	11 (35.5)
	Moderate	6 (19.4)
	High	14 (45.2)
PSQI	Subjective sleep quality	0.83 ± 0.6
	Sleep latency	1.5 ± 1.02
	Sleep duration	0.54 ± 0.8
	Sleep efficiency	0.67 ± 1.1
	Sleep disturbance	1.1 ± 0.5
	Use of sleep medication	0.29 ± 0.6
	Daytime dysfunction	1.09 ± 0.7
	Global score	6.1 ± 2.5
WHOQOL-BREF – Physical	Low	6 (12.9)
	Moderate	0 (0)
	High	25 (80.6)
WHOQOL-BREF – Psychological	Low	4 (12.9)
	Moderate	3 (9.7)
	High	24 (77.4)
WHOQOL-BREF – Social	Low	9 (29)
	Moderate	2 (6.5)
	High	20 (64.5)
WHOQOL-BREF – Environment	Low	4 (12.9)
	Moderate	1 (3.2)
	High	26 (83.9)

Data is presented as mean ± standard deviation and number (percentage); y = years, cm = centimeters; kg = kilograms; BMI = Body mass index; IPAQ-SF = International Physical Activity Questionnaire-Short Form; PSQI = Pittsburgh Sleep Quality index; WHOQOL-BREF = World Health Organization Quality of Life-BREF.

Table 2 summarizes posture-specific and composite RULA scores for the cohort. Across segments, postural risk clustered in the mid-range. Upper-limb components were most often scored 2–3 (upper arm, lower arm, wrist), while wrist twist was predominantly neutral (score 1) and legs were almost universally stable (score 1). Neck and trunk tended to fall in the middle categories (mostly 2–3). Composite upper-limb risk (Score A/C) concentrated at 3–4, and axial composites (Score B/D) were similarly centered at 2–4. The Grand RULA score was chiefly 3–4 (74.2%), with a smaller proportion at 5–6 (22.6%), indicating that most students required investigation and possible changes, and a notable minority warranted prompt ergonomic action.

**Table 2:** Distribution of RULA item and composite scores among the sample.

Items	Rula Score					
	1	2	3	4	5	6
Upper arm posture	3 (9.7)	15 (48.4)	10 (32.3)	3 (9.7)		
Lower arm posture		17 (54.8)	14 (45.2)			
Wrists posture	6 (19.4)	10 (32.3)	15 (48.4)			
Wrist twist posture	28 (90.3)	3 (9.7)				
Score A		1 (3.2)	15 (48.4)	13 (41.9)	2 (6.5)	
Score C		1 (3.2)	8 (25.8)	14 (45.2)	8 (25.8)	
Neck posture	3 (9.7)	15 (48.4)	13 (41.9)			
Trunk posture	2 (6.5)	20 (64.5)	9 (29)			
Leg posture	29 (93.5)	2 (6.5)				
Score B		13 (41.9)	9 (29)	9 (29)		
Score D		1 (3.2)	13 (41.9)	10 (32.3)	5 (16.1)	2 (6.5)
Grand RULA score		1 (3.2)	13 (41.9)	10 (32.3)	5 (16.1)	2 (6.5)

Date is presented as number (percentage); RULA = Rapid Upper Limb Assessment; Score A= upper arms, lower arms, and wrists postures; Score B = neck, trunk, leg postures; Score C = Score A + muscle use and force scores for group A; Score D = Score B + muscle use and force scores for group B.

Kruskal–Wallis tests examined differences in RULA outcomes across PA levels (IPAQ-SF: low, moderate, high). Upper arm posture, Score A (upper-limb composite), and Score C (Score A + muscle/force adjustments) differed by PA: Χ^2^ (2) = 6.07, *p* = 0.04, ε^2^ = 0.20; Χ^2^ (2) = 7.69, *p* = 0.02, ε^2^ = 0.25; and Χ^2^ (2) = 6.35, *p* = 0.04, ε^2^ = 0.21, respectively. Medians (IQR) indicated a consistent pattern in which the moderate PA group showed the least favorable scores relative to low PA and high PA. No significant differences were observed for Lower arm, Wrist, Wrist twist, Neck, Trunk, Leg, Score B/D, or the Grand RULA (all Χ^2^ (2) ≤ 3.72, *p* ≥ 0.05, ε^2^ ≤ 0.12). Overall, habitual activity was most closely related to upper-limb risk components (Score A/C), whereas neck–trunk–leg postures and the Grand RULA appeared less sensitive to PA in this sample (Table 3).

**Table 3:** The RULA scores of the sample are divided by the physical activity levels.

RULA score	Physical Activity level			Χ^2^ (2)	*p*	ε²
	Low	Moderate	High			
Upper arm posture	2 (0.50)	3 (0.75)	2 (1)	6.07	0.04*	0.20
Lower arm posture	2 (1)	3 (0.75)	2 (1)	1.44	0.48	0.04
Wrists posture	2 (1)	3 (0)	2 (1.75)	3.72	0.15	0.12
Wrist twist posture	1 (0)	1 (0)	1 (0)	2.65	0.26	0.08
Score A	3 (1)	4 (0)	3 (1)	7.69	0.02*	0.25
Score C	4 (0.5)	5 (0.75)	4 (1)	6.35	0.04*	0.21
Neck posture	2 (1)	2.5 (1)	2 (1)	0.46	0.79	0.01
Trunk posture	2 (1)	2 (0)	2 (1)	3.29	0.19	0.10
Leg posture	1 (0)	1 (0)	1 (0)	1.74	0.41	0.05
Score B	2 (1)	3 (0.75)	3 (2)	1.56	0.45	0.05
Score D	3 (1.5)	3.5 (1)	4 (1.75)	1.83	0.40	0.06
Grand RULA score	3 (1)	4 (0.75)	4 (1.75)	2.93	0.23	0.09

Date is presented as median (IQR); RULA = Rapid Upper Limb Assessment; Score A= upper arms, lower arms, and wrists postures; Score B = neck, trunk, leg postures; Score C = Score A + muscle use and force scores for group A; Score D = Score B + muscle use and force scores for group B; * indicates significant.

**Fig. 2:**
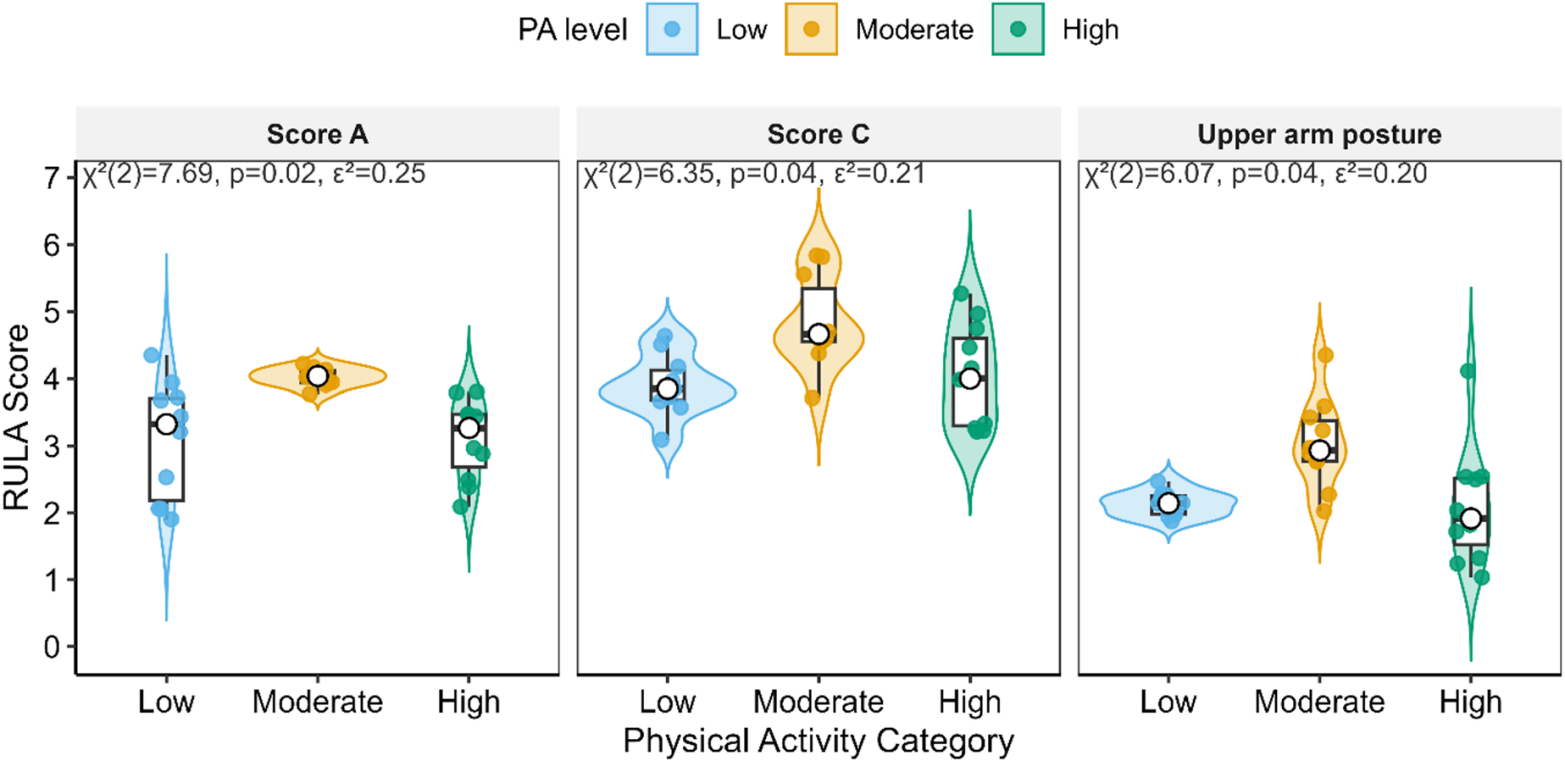
RULA outcomes by physical activity (IPAQ-SF) category for variables with significant between-group differences. Violin plots (with overlaid boxplots showing median and IQR) and jittered points (individual participants) depict distributions for Upper arm posture, Score A (upper-limb composite), and Score C (Score A + muscle/force adjustments) across Low, Moderate, and High PA. Lower values indicate more favorable posture.

As shown in Fig. 2, the RULA outcomes that differed across PA categories—Upper arm posture, Score A, and Score C—exhibited a consistent pattern: the moderate PA group tended to have higher (less favorable) scores, whereas the high PA group showed lower (more favorable) scores. Distributions (medians, IQRs, and individual data points) further indicate that these differences are not driven by outliers but reflect a shift in the central tendency across PA levels.

In ordinal (proportional-odds) models (Table 4), only the wrist-posture model showed evidence of overall fit (AIC = 41.8; R^2^ = 0.30; χ^2^ (5) = 13.10, *p* = 0.02). Within this model, BMI was the sole significant predictor: higher BMI was associated with greater odds of being classified in a worse wrist-posture category (OR = 1.33, 95% CI [1.02, 1.73], *p* = 0.03), after adjustment for PSQI, overall QoL, and physical-activity (IPAQ) category. The Score A (upper-limb composite) and upper-arm posture models did not show meaningful improvement over intercept-only specifications (Score A: AIC = 35.9; R^2^ = 0.07; X^2^ (5) = 1.67, *p* = 0.89; Upper arm: AIC = 48.0; R^2^ = 0.14; χ^2^ (5) = 6.20, *p* = 0.28). For these outcomes, individual predictors—including PA contrasts and PSQI—were not statistically significant, and several estimates exhibited wide CIs, indicating limited precision with the current sample. Overall, the pattern suggests a specific anthropometric contribution to wrist-posture classification, with inconclusive evidence for PA, sleep quality, or QoL across the other RULA domains.

**Table 4:** Ordinal (proportional-odds) regression predicting RULA scores (odds ratios with 95% CIs).

Predictor	OR	95% CI	*p*
1) Outcome: Score A			
BMI (kg/m²)	0.87	[0.58–1.12]	0.40
PSQI (global)	0.44	[0.44–1.57]	0.75
IPAQ: Low vs. High	1.48	[0.04–33.61]	0.79
IPAQ: Moderate vs. High	2.94	[0.09–50.69]	0.48
QoL (overall)	0.45	[0.03–3.11]	0.47
Model fit	AIC = 35.9; R^2^ = 0.07; χ^2^ (5) = 1.67, *p* = 0.89		
2) Outcome: Upper arm posture			
BMI (kg/m²)	0.96	[0.82–1.13]	0.63
PSQI (global)	1.05	[0.76–1.44]	0.75
IPAQ: Low vs. High	0.67	[0.11–4.07]	0.66
IPAQ: Moderate vs. High	0.98	[0.81–35.27]	0.07
QoL (overall)	0.80	[0.29–2.22]	0.67
Model fit	AIC = 48; R^2^ = 0.14; χ^2^ (5) = 6.20, *p* = 0.28		
3) Outcome: Wrist posture			
BMI (kg/m²)	1.33	[1.02–1.73]	0.03*
PSQI (global)	1.40	[0.89–2.22]	0.13
IPAQ: Low vs. High	0.33	[0.03–3.03]	0.33
IPAQ: Moderate vs. High	14.77	[0.72–47.66]	0.08
QoL (overall)	1.49	[0.40–5.55]	0.54
Model fit	AIC = 41.8; R^2^ = 0.30; χ^2^ (5) = 13.1, *p* = 0.02*		

RULA = Rapid Upper Limb Assessment; Score A= upper arms, lower arms, and wrists postures; B = unstandardized coefficient; β = standardized beta coefficient; BMI = Body Mass Index; PSQI = Pittsburgh Sleep Quality Index; IPAQ = International Physical Activity Questionnaire; QoL = Quality of Life; CI = Confidence Interval; Reference group for IPAQ contrasts = High PA; * indicates significant.

**Fig. 3:**
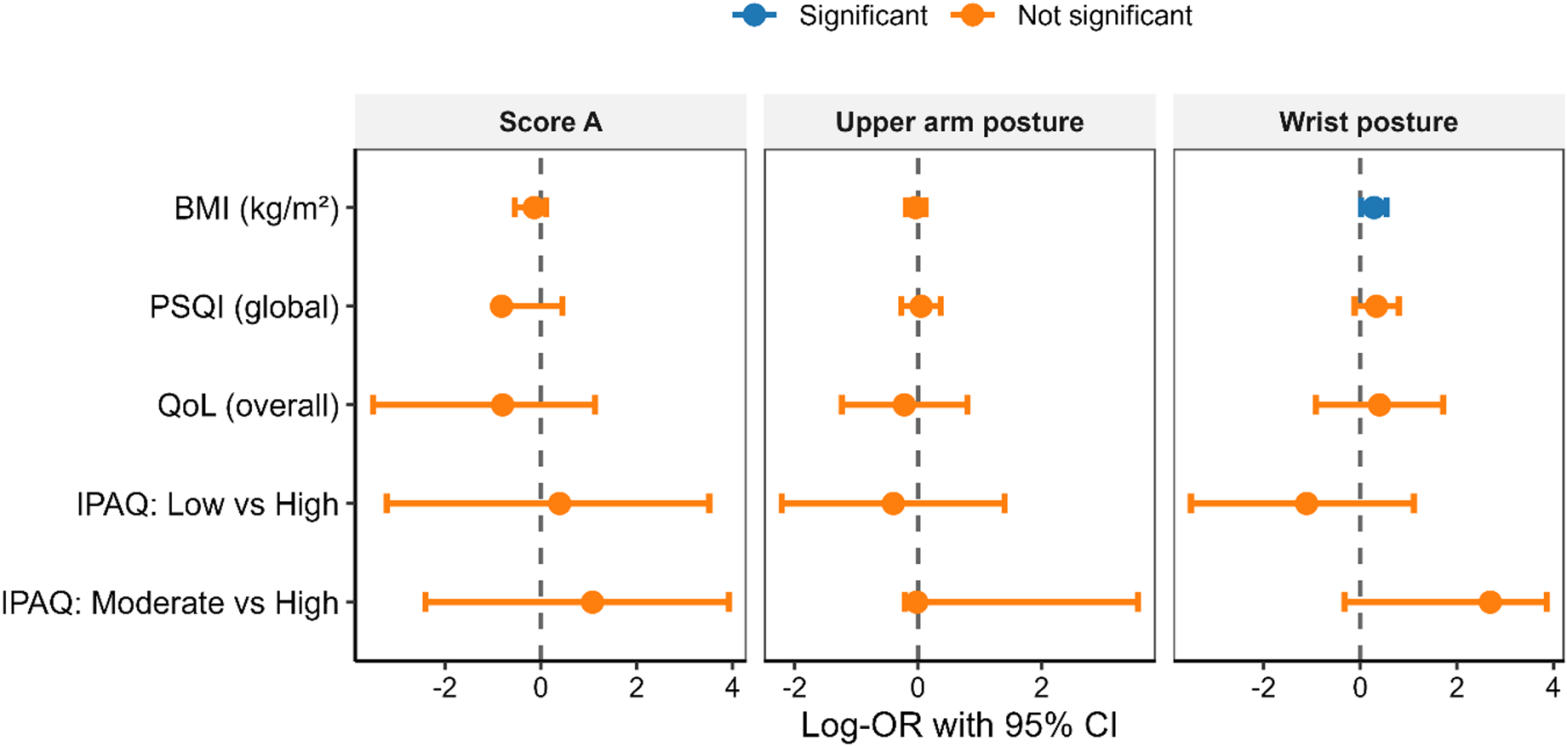
Forest plot of ordinal regression coefficients (log-odds) predicting RULA outcomes. Points show the log-OR with 95% CIs for each predictor; the dashed vertical line at 0 indicates no association. Panels display (left to right): (**A**) Score A (upper-limb composite: upper arm, lower arm, wrist), (**B**) Upper arm posture, and (**C**) Wrist posture. Predictors include BMI (kg/m²), PSQI global score (sleep quality), overall QoL, and physical activity contrasts from IPAQ-SF (reference = high PA; low vs. high; moderate vs. high). Blue denotes statistically significant effects (*p* < 0.05); orange denotes non-significant effects. Positive values indicate higher (worse) RULA scores; negative values indicate lower (better) scores.

As summarized in Fig. 3, the multivariable ordinal (proportional-odds) models for three RULA outcomes—Score A (upper-limb composite), Upper-arm posture, and Wrist posture—plotted as log-odds coefficients (points) with 95% CIs (bars). The reference line at 0 denotes no association. Consistent with the tabulated results, only BMI was a statistically significant predictor for Wrist posture (OR > 1), indicating higher BMI was associated with greater odds of being in a worse wrist-posture category. PSQI (sleep quality), overall QoL, and the IPAQ contrasts (Low vs. High; Moderate vs. High, reference = High PA) were not significant in the Score A or Upper-arm models, and their CIs generally crossed the null in all panels.

## Discussion

This study quantified task-level ergonomic risk in medical laboratory students and examined its associations with lifestyle and anthropometric factors. RULA scoring showed that most participants clustered in mid action levels (Grand RULA predominantly 3–4), indicating postures warranting investigation and possible modification. In non-parametric comparisons across PA strata, upper-limb risk differed for selected components—upper-arm posture and the composite upper-limb indices (Score A and Score C)—with more favorable scores generally observed in the high-activity group, whereas lower-arm, wrist-twist, and axial composites (Score B/D) did not differ. In multivariable ordinal models, higher BMI was associated with greater odds of worse wrist-posture categories; other predictors (sleep quality, overall QoL, and PA contrasts) were not consistently related to Score A or upper-arm posture after adjustment. Collectively, these findings highlight the multifactorial nature of posture-related ergonomic outcomes and support an integrated approach to risk reduction that considers both physical and lifestyle-related factors. Because assessments were conducted under standardized tasks over a brief observation window, results should be interpreted as between-participant variability in postural strategies rather than evidence of causal effects or underlying mechanisms; experimental or longitudinal studies are needed to determine whether modifying these factors leads to meaningful changes in RULA-defined risk during laboratory activities.

Recent evidence indicates that laboratory learning environments expose students to meaningful ergonomic risk—most notably from prolonged microscopy, repetitive pipetting, and static or awkward postures. In an Australian cross-sectional survey of medical science students, over one-third reported a laboratory-related musculoskeletal problem in the prior 12 months and just over one-fifth in the prior 7 days, with the lower back, neck, and upper back most affected—patterns that mirror those seen in professional laboratory staff^10^. Comparable findings emerged in a Pakistani cohort of biomedical science students, where lower back, neck, and shoulder complaints predominated and were associated with gender, predominant work position, and specific laboratory activities^9^.

Broader surveys of allied health undergraduates likewise document substantial symptom burdens (e.g., 12-month neck symptoms [41.6%] and any-site symptoms [73.6%]), underscoring that students are not exempt from ergonomic stressors^55^. Postural appraisals using RULA corroborate elevated risk during student practical’s; a university-based assessment of postures in computer laboratories, mechanical workshops, and other settings reported RULA scores approaching the maximum (~ 7), indicating an urgent need for investigation and change^56^. In contrast, our cohort’s RULA distribution placed most medical laboratory students in the low- to medium-risk range, suggesting that while laboratory tasks are plausibly hazardous, risk magnitude varies by task mix, workstation configuration, and cohort characteristics, and may be reduced through targeted ergonomic controls and training.

Task analyses from laboratory ergonomics and occupational studies consistently implicate pipetting, microscopy, and prolonged sitting/standing as drivers of upper-quarter and spinal loading, mirroring symptom distributions reported by students^57^. Although student-specific prevalence data from the Middle East are limited, studies of clinical laboratory personnel in Saudi Arabia indicate high 12-month musculoskeletal disorder prevalence (71%), situating laboratory ergonomic risk along the training-to-workforce continuum^7^. Comparable patterns have been observed in broader student cohorts and early-career laboratory staff, suggesting that postural loading begins during training and parallels exposures later seen in professional roles^58,59^. In biomedical laboratory workers specifically, epidemiological and ergonomic investigations identify pipette use as salient exposure, with upper-limb complaints influenced by device design and task characteristics^60^.

Evidence indicates that PA mitigates posture-related risk: exercise programs lower neck-pain incidence and symptoms in desk-based workers and breaking up sitting reduces neck/shoulder and general musculoskeletal complaints^61–63^. Consistent with this, our cohort’s higher PA was associated with more favorable upper-arm posture and lower upper-limb composites (Score A and Score C). Broader task-level analyses integrating RULA with PA or symptom data likewise suggest that greater habitual PA corresponds to fewer posture-related complaints, whereas low PA and prolonged sedentary behavior are linked to higher symptom prevalence^32,64,65^. These findings support embedding PA promotion and movement breaks within laboratory curricula to reduce ergonomic risk.

A substantial body of longitudinal and cross-sectional research implicates sleep quality as a proximal determinant of musculoskeletal pain with evidence for both bidirectionality; sleep disturbance predicting incident/persistent neck and other musculoskeletal disorder pain, and pain subsequently degrading sleep, and mediation; sleep partially transmitting the effects of adverse work organization and ergonomic exposures to pain and functional limitation^30,66^. In parallel, systematic reviews indicate that WRMSDs are associated with reduced health-related QoL across occupational sectors, and student/early-career samples similarly demonstrate QoL decrements in the presence of musculoskeletal disorders^33,67^. In our cohort, poorer sleep showed a no association with less favorable upper-limb posture (e.g., wrist/upper-arm metrics), while fatigue-related mechanisms (e.g., reduced proprioceptive acuity or fine-motor control) remain plausible, the present data do not substantiate a measurable association; larger, adequately powered studies with objective sleep measures (e.g., actigraphy) are warranted.

Moreover, WHOQOL-BREF domains were not significantly associated with RULA-defined risk. This apparent discrepancy with the broader health-related QoL literature is plausibly explained by ceiling effects in a young, generally healthy student sample; construct specificity (WHOQOL-BREF is broad and not posture-targeted); lower symptom severity and shorter exposure duration in laboratory training relative to full-time work; and limited statistical power in a small, single-site study. Collectively, prior evidence and our findings suggest that sleep quality is a proximal, modifiable correlation of posture-related risk in student laboratory tasks, whereas global QoL contributes little incremental variance after accounting for sleep, PA, and anthropometry in early training contexts.

### Practical and educational implications

The observed concentration of risk in upper-limb segments supports a dual-track intervention model. Environmental controls should prioritize adjustable bench and microscope eyepiece heights, provision of forearm support, neutral-wrist grips for fine-motor tasks, task rotation to reduce static loading, and programmed micro-breaks (30–60 s every 20–30 min). Educational measures should include a brief, RULA-informed posture screening with immediate feedback; a standardized self-setup checklist (seat height yielding ~ 90° elbow flexion, eyepiece aligned to eye level, forearm supported, wrist neutral); and concise modules on workstation configuration and symptom self-monitoring. Given associations between posture and lifestyle factors, programs should encourage activity targets consistent with IPAQ-SF “high” categories and direct students with PSQI > 5 toward sleep-hygiene resources. At the policy level, departments should adopt procurement standards (adjustable furniture and arm supports), require an ergonomic readiness sign-off at the start of practical blocks, and conduct termly audits using Grand RULA Action Levels and brief symptom surveillance, with targeted coaching for higher-risk subgroups (e.g., elevated BMI and weight, low PA, poor sleep). The association between BMI/weight and higher RULA scores is consistent with established ergonomics evidence regarding anthropometric mismatch and workstation fit. We therefore interpret this result as confirmatory rather than the primary novel contribution of the study.

### Strength and limitations

This study offers task-specific, segment-level profiling of student laboratory work using standardized video capture and RULA scoring, yielding interpretable Action Levels with direct implications for teaching and workstation redesign. Validated Arabic versions of the IPAQ-SF, PSQI, and WHOQOL-BREF were used with clear scoring procedures. Nonetheless, external validity is limited by the small, single-institution, male-only convenience sample and the cross-sectional, observational design. RULA reflects posture at the time of observation and does not directly quantify exposure dimensions such as duration, repetition, or force, which may be central determinants of musculoskeletal risk. The 5-minute observation window may underestimate cumulative axial fatigue and longer-term postural drift; several macro-ergonomic determinants were not quantified (e.g., detailed workstation geometry, exposure duration, daily hours, prior ergonomics training, repetition/contact stress); a potential observer effect resulting from the video recording method utilized; and lifestyle measures (PA, sleep, QoL) were self-reported, therefore, findings should be interpreted as preliminary. Analyses were exploratory, increasing the risk of Type I error; we therefore foreground effect sizes and CIs and recommend cautious interpretation.

Future work should recruit larger, mixed-gender, multisite cohorts; extend observation periods; incorporate objective sensing (e.g., EMG, actigraphy) and comprehensive workstation audits; and prospectively evaluate curriculum-embedded ergonomic and wellness interventions, including ergonomics training and deployment of adjustable workstations. In addition, experimental and/or repeated-measures designs that manipulate workstation geometry and training exposure, while capturing objective exposure metrics (e.g., duration, repetition, force) alongside posture scoring, are needed to determine whether the observed associations reflect truly modifiable risk factors and to inform integrated prevention strategies that combine workstation audits, education, and organizational determinants.

## Conclusion

Upper-limb ergonomic risk emerged as the principal concern during laboratory tasks, with BMI showing significant, actionable associations and PA and sleep quality exhibiting limited effects. Academic programs can respond by implementing adjustable benches and eyepiece heights, forearm support, task rotation, and scheduled micro-breaks, alongside brief modules on PA and sleep hygiene within laboratory teaching. Given design and sampling constraints, generalization should be cautious; multisite, mixed-gender studies that use objective sensing and detailed workstation audits are warranted to guide policy and procurement for student laboratories. Overall, the results support early ergonomic education and targeted preventive strategies to protect musculoskeletal health during training and beyond.

## Data Availability

Data will be available upon request from the corresponding author.
